# Language as a cue for social categorization in bilingual communities

**DOI:** 10.1371/journal.pone.0276334

**Published:** 2022-11-02

**Authors:** Anna Lorenzoni, Mikel Santesteban, Francesca Peressotti, Cristina Baus, Eduardo Navarrete

**Affiliations:** 1 Dipartimento di Psicologia dello Sviluppo e della Socializzazione, Università degli Studi di Padova, Padua, Italia; 2 Department of Linguistics and Basque Studies, University of the Basque Country (UPV/EHU), Leioa, Spain; 3 Department of Cognition, Development and Educational Psychology, University of Barcelona, Barcelona, Spain; CNRS - Université d’Aix-Marseille, FRANCE

## Abstract

This registered report article investigates the role of language as a dimension of social categorization. Our critical aim was to investigate whether categorization based on language occurs even when the languages coexist within the same sociolinguistic context, as is the case in bilingual communities. Bilingual individuals of two bilingual communities, the Basque Country (Spain) and Veneto (Italy), were tested using the memory confusion paradigm in a ‘*Who said what*?*’* task. In the encoding part of the task, participants were presented with different faces together with auditory sentences. Two different languages of the sentences were presented in each study, with half of the faces always associated with one language and the other half with the other language. Spanish and Basque languages were used in Study 1, and Italian and Venetian dialect in Study 2. In the test phase, the auditory sentences were presented again and participants were required to decide which face uttered each sentence. As expected, participants error rates were high. Critically, participants were more likely to confuse faces from the same language category than from the other (different) language category. The results indicate that bilinguals categorize individuals belonging to the same sociolinguistic community based on the language these individuals speak, suggesting that social categorization based on language is an automatic process.

## Introduction

Categorization is a fundamental human cognitive process that has the function of organizing and processing stimuli quickly and automatically [1–3]. As human beings, each of us belongs to different social categories: we can be categorized, for instance, as young or old, sporty or non-sporty, parents or non-parents. Social categorization refers to the tendency to classify individuals in terms of the categories they belong or do not belong to. Social categorization is an automatic phenomenon that occurs when we meet a new person and can influence the way we perceive people from different groups [4–6]. Decades of research have been devoted to the study of race, age and gender as the three major cues of social categorization [7–12]. Here we focus on another cue that has received less attention. This is the case of the language used by the interlocutor, which remains unknown until she or he starts speaking. Although people may be able to guess which language is spoken by the interlocutor based on the sociolinguistic contexts they live in, as for instance which language is more frequently used in that context, these guesses can be incorrect. Thus, the language of the interlocutor will only be known (and the guesses confirmed or disconfirmed) when the interlocutor speaks.

Recent studies have shown that infants use language to encode individuals in different groups according to the language they speak. For instance, Kinzler, Dupoux and Spelke [13] observed that 6-month old infants prefer looking at speakers of their same native language than those who speak a different language. Other studies reported that 11- and 19-month old infants, when learning new information, look more frequently at members belonging to the same linguistic group than at people of a different linguistic group [14–16]. These results with the language cue would be analogous to what has been observed with other cues, such as race and gender [17,18].

Empirical investigations on the role of language as a cue for categorization in adults focused initially on accent, that is, the peculiar pronunciation of a group of individuals from a particular region. Pietraszewski and Schwartz ([19], see also [20]) have exploited the logic underlying the memory confusion paradigm [21,22], whereby, if an individual’s feature is a cue for categorization, then individuals sharing this feature will be more likely to be confused between each other than between individuals not sharing this feature. In their study, participants were first exposed to pairings of faces and audio statements. Half of the statements were uttered in an English accent (e.g., American accent) and the other half in a different English accent (e.g., British accent). After a brief distractor task, participants were asked to determine which speaker made each statement by selecting the appropriate face from an array containing all the faces. The results showed that when participants incorrectly attributed statements to speakers, they were more likely to choose a speaker with the same accent as the original speaker. That is, participants made more same-accent errors, confusing speakers from the same accent category, than between-accent errors, confusing speakers from the different accent category. These results were interpreted as evidence that accent is a cue for automatic and implicit categorization of faces.

In a recent study Baus, Ruiz-Tada, Escera & Costa [23] have replicated this finding with two different languages instead of two different accents of the same language. Specifically, Spanish participants were exposed to Spanish and English statements. Similar to what was obtained by Pietraszewski and Schwartz [19], same-language errors were more frequent than between-language errors. Interestingly, Baus and colleagues further measured the electrophysiological activity associated to language categorization in an oddball paradigm. The ERP analysis showed an early visual mismatch negativity (vMMN) for between-language category faces, but not for within-category faces. This result seems to indicate that language categorization influences the early stages of face processing. In sum, findings from the memory confusion paradigm suggest that people group individuals (i.e. faces) according to the language (or accent) they speak. Moreover, at the neural level, such categorization is an automatic process able to modulate early visual perceptual processing. The present study aims to define the boundaries of this phenomenon.

One common feature of the studies conducted so far refers to the fact that the accents or languages used in the studies belonged to two different sociolinguistic contexts. For instance, participants in Pietraszewski and Schwartz’s studies were American citizens from California who were tested with different English accents, including American, British, or Irish. Thus, the accents tested belonged to two different communities, in this case, two English-speaking countries. Similarly, in the study by Baus and colleagues, participants were Spanish dominant, had English as a foreign language and belonged to a sociolinguistic community where Spanish is an official language while English is not. It is therefore possible that participants are not only categorizing faces according to the accent or language they speak, but they are also categorizing faces mediated by the different sociolinguistic communities to which these faces could be ascribed. Some empirical findings would be congruent with this possibility. It is known that foreign accent generates an immediate classification of the speaker as an out-group member and that such classification activates the stereotypes and stigmas associated to this group [24–28]. Therefore, participants could classify speakers according to the accent or language they speak and/or the stereotypes associated. At the same time, some studies have suggested a role of this kind of social stereotypes on speaker recognition [29–31]. The main aim of the present study was to explore whether language categorization is an automatic phenomenon occurring even when the languages associated to the stimuli (i.e., faces) cannot be ascribed to different social communities. To do this we tested bilingual communities.

People living in a bilingual community are regularly exposed to both single and dual language interaction contexts. More critical for our purposes, an individual from this community may be associated with the two languages used in the community rather with a single language. That is, unlike what normally occurs in monolingual communities where there may be a one-to-one correspondence between interlocutor and language, in bilingual communities there may be a one-to-two correspondence. Interestingly, bilingual speakers seem to be sensitive to this correspondence. Recent studies have shown that bilinguals are able to adapt to language-contexts based on prior knowledge about interlocutors. For instance, Molnar, Ibáñez-Molina and Carreiras [32] familiarized Basque-Spanish bilinguals with three different interlocutors who spoke Spanish, Basque, or both languages. Immediately after the familiarization, participants completed an audio-visual lexical decision task in which the interlocutors produced target words in Spanish or Basque. Reaction times were faster when the language the interlocutors spoke at the lexical decision task matched the language used during familiarization with respect to when the language did not match. In an event-related potential adaptation of Molnar at al.’s study, Martin, Molnar and Carreiras [33] observed that faces associated to one language (i.e., monolingual speakers) elicited a larger early negativity ERP component compared to those associated with two languages (i.e., bilingual speakers). The difference in the ERP deflection was reliable even before the speaker started to speak, suggesting that faces might convey information pertaining to the language(s) associated with the face. These studies suggested that bilinguals are able to anticipate which language their interlocutor will use, congruent with some models of bilingual language control [34,35].

In the present study, we test whether language automatically functions as a cue for face connotation, even in conditions in which language does not clearly distinguish between different social groups (i.e., when the languages at test belong to the same sociolinguistic context). Participants were bilingual speakers living in a bilingual community, who are exposed daily to the two languages of their community. We took advantage of the memory confusion paradigm. If language categorization is an automatic process, we expected to replicate previous findings and observe more same-language errors than different-language errors; that is, when participants make an error attributing a statement to a speaker, they are expected to be more likely to choose a speaker of the same language. By contrast, if language categorization is contingent on sociolinguistic categorization, the effect should appear only when languages are ascribed to different social groups, as was the case in the studies by Pietraszewski and Schwartz [19], and Baus et al [23]. Under this latter hypothesis, no language categorization effect should be expected in our studies, where the languages used belong to the same sociolinguistic context in which the bilingual participants are exposed daily to faces speaking those languages.

To obtain a better description of the categorization role of language within bilingual contexts, we tested two different types of bilingual communities. In the first study, we tested Spanish and Basque, two typologically different languages: Spanish is a Romance language from the Indo-European language family while Basque is a non-Indo-European language isolate [36,37]. Both are co-official languages in the Basque Autonomous Community and Navarra (northeastern Spain). In the second study, we tested two varieties of the Romance language family: Italian and the Veneto dialect [38,39]. The Veneto dialect is a non-official regional language spoken in Veneto, a northeastern region of Italy, where the only official language is Italian ([38,40]; see also [41]). It is possible that in these communities the use of a specific language is associated with different cultural and political sensitivities. For example, the use of Spanish, or Basque, could indicate that the speaker has a different group identification with respect to Spanish and Basque identities; the same situation could happen in relation to the use of Italian or Venetian. If this were the case, instead of, or in addition to language, the participant’s cultural and political sensitivities towards each language could drive the categorization of speakers in our experimental paradigm. To control the impact of this variable, we used a group identification scale to ensure that our participants were neutral or positive towards Spanish and Basque identities (Study 1) and towards Italian and Venetian identities (Study 2).

A second goal of the current research was to explore whether the language effect on face categorization is modulated by the degree of bilingualism which we operationalize as the amount of participant’s exposure to each of the two languages. In their study, Molnar and colleagues [32] tested two groups of Spanish-Basque bilinguals. One group was composed of balanced (highly proficient) bilinguals who acquired Basque before the age of 3 and reported using both languages on a daily basis with family, friends, and colleagues. The other group was composed of unbalanced (less proficient) Spanish-Basque bilinguals who started learning Basque in school-settings between the age of 9 and 14 and reported using Spanish as the primary language for daily communication. Only balanced bilinguals showed adaptation of their language comprehension processes to the linguistic identity of the interlocutor. Such an effect was not observed in the unbalanced bilinguals’ group. To explore the extent to which language exposure affects the language categorization phenomenon, we estimated the relative use of each language for each participant and we added this measure as a continuous predictor to the main analysis (see for a similar procedure, [42]).

In sum, as a main hypothesis, more same-language errors than different-language errors were expected in the two populations of bilinguals. Such a result would be congruent with the assumption that language categorization is an automatic process. In addition, in further analyses we explored whether language exposure in daily social interactions modulated this effect.

## Study 1: Spanish-Basque bilinguals

### Materials and methods

The study was preregistered as a Research Report Protocol [43]. All experimental procedures were approved by the local Research Ethics Committees of the University of Padova (Protocol number: 3589; Title: *The social bilingual brain*). Material, data and scripts for analysis can be consulted on the platform OSF (https://osf.io/3fudg/).

#### Participants

50 Spanish-Basque bilingual participants took part in Study 1 (31 female). All participants were required to give written informed consent.

#### Materials

Eight gray-scale photographs of male Caucasian faces were taken from Martinez & Benavente [44]. All of them were emotionally neutral and had no extra visual details. Twenty-four non-autobiographical sentences were created and then recorded in Spanish and Basque using the software Audacity (v 2.0.3) (e.g., *La tienda se queda vacía*–*Denda hutsik geratu da*; “The store becomes empty”, in Spanish and Basque, respectively). The differences in length between Spanish and Basque sentences were measured by calculating the number of phonemes because words are not a good unit for comparing Spanish and Basque. This is because Basque is an agglutinative language, and all determiners and prepositions are embedded with their nouns, while in Spanish determiners and prepositions are separated. The number of phonemes did not diverge between Spanish [mean = 19.58 phonemes, range = 13–25] and Basque [mean = 20 phonemes, range = 12–22] (t < 1) sentences. Recording durations for sentences in Spanish [mean = 1.91 seconds, range = 1.52–2.48] and Basque [mean = 1.84 seconds, range = 1.05–2.49] did not differ (t(46) = 0.79, p = .42). Four male native Spanish speakers and four male native Basque speakers recorded the sentences. The final design consisted of photographs of faces accompanied by a voice speaking either in Spanish or in Basque. Sixteen lists were created to counterbalance the face, sentence and language. Therefore, all faces accompanied every sentence in both languages across all participants.

#### Procedure

**Fig 1:**
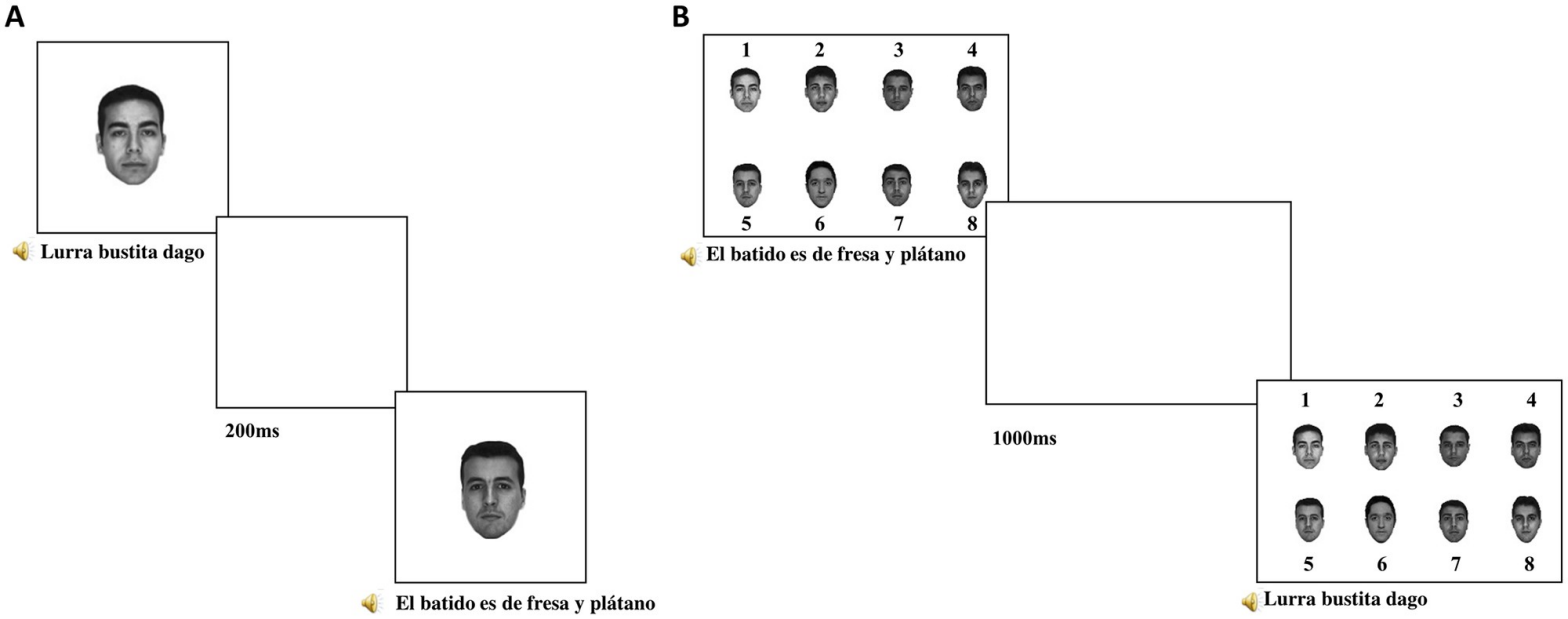
The procedure of the memory confusion paradigm. This diagram shows the two main phases of the paradigm. On Panel A, the encoding phase, where faces were presented with the audio sentences. On panel B, the final recognition phase. Grayscale photos of eight Caucasian males with neutral expressions were selected from the free AR face database [44].

The experiment consisted of four parts: an encoding phase, a distractor task (*tetris game*), a recognition phase and a questionnaire (see below). At the beginning of the experiment, the participant was only aware of the first phase and was informed that the study will take approximately 15 minutes. In the initial encoding phase, photographs of faces were presented on the screen one at a time along with the auditory presentation of the sentences. Participants were only asked to form impressions about the speakers as they watched and listened because later they were going to be asked questions about them. Trial structure was the following: one photo and audio were presented simultaneously on the screen. Each speaker’s photo was displayed for the entire duration of the statement, plus two additional seconds thereafter, followed by a blank presented on the screen for 200ms (Fig 1). Each of the 8 faces was presented 3 times during the encoding phase, for a total of 24 presentations. The three presentations of each face had three different sentences, but the voice was the same. In other words, each face was paired with the same voice and was associated with three different sentences. The language of the sentences in the first two positions was counterbalanced between the lists so that 8 lists started with two Spanish sentences and 8 with two Basque sentences. Language order was unsystematic thereafter, within the constraint that each speaker spoke once during statements 1–8, once again in statements 9–16, and once in statements 16–24. Upon completion of the encoding phase, participants were engaged in a distractor filler task (Tetris game) for 2 minutes to avoid having the recognition phase immediately after the encoding phase.

After that, participants started the second phase of the memory confusion paradigm, the recognition phase, in which all 8 photographs were presented on the screen, numbered from 1 to 8. Face order was randomized across trials. Then, the same 24 sentences of the encoding phase were presented again in auditory form. The participant decided which of the 8 faces accompanied the sentence in the encoding phase by clicking on the corresponding number. The eight faces remained on the screen until the participant’s response, after which a blank screen lasting 1000 ms was presented (see Fig 1). This procedure continued until all 24 sentences of the encoding phase were presented. The experiment lasted about fifteen minutes.

After the recognition phase, participants completed the questionnaire which consisted of four parts: a) *general information* concerning the language the participant used as a child and the age of acquisition; b) *perceived proficiency*, in which the participant rated his/her degree of perceived proficiency in comprehension and production using a 1–10 point scale (1 = “none”; 10 = “perfect”) in both languages; c) *language use*, in which the participant quantified the use of each language in various daily activities; and d) *group identification*, where the participant’s level of identification with their groups (i.e., Spanish and Basque, or Italian and Venetian for Study 1 and Study 2, respectively) was assessed in 4 questions using a 1–7 point scale (1 = “not at all”; 7 = “very much”). These questions were based on research by Latrofa, Vaes, Pastore & Cadinu [45]. In order to ensure that our participants were highly proficient and able to interact in both languages, only results of those participants with a mean >6 in part b of the questionnaire (*perceived proficiency)* in both languages were analysed. A Relative Use Index was calculated for each participant applying the following formula to the daily activities answered in part c of the questionnaire (*language use)*: (value in language A–value in language B) / (value in language A + value in language B). The mean between the scores obtained in all daily activities corresponds to the Relative Use Index for a particular individual. This ratio will score from -1 to 1. The value of 0 indicates a perfectly balanced bilingual, that is, with a similar amount of use of the two languages. Positive or negative values indicate the inclination of use towards one language or the other.

At the end of the experimental session participants were thanked and debriefed by describing the real aims of the experiment. In addition, participants were again asked for their consent for their results to be used.

#### Methodology for data collection

The experiment took place online, through the *PCIbex* platform [46]. Participants were to access the test by clicking on a link. Participants were recruited through the participant pool database of The Bilingual Mind research group (https://www.ehu.eus/HEB/) of the University of Basque Country.

#### Methodology for analysis

First, to test for the presence of a Language effect, categorization was measured on a participant basis by calculating the difference in error rates between same-language errors and different-language errors. While there are only three possibilities to make same-language errors (because one of the faces is the correct answer), there are four possibilities to make a different-language error. To correct for this discrepancy, the number of different-language errors was multiplied by 0.75. Following previous studies that have used this paradigm [19,20], paired t-test analyses were performed between same-language and different-language errors (see [47] for validation of this method). Different to what was declared in the protocol, to explore the influence of language exposure on the language categorization effect, the Relative Use Index was added as a fixed effect in a linear model. As a sanity check to control whether the memory confusion paradigm is doing what it is supposed to do, we expected error rates to be high. In particular, according to the previous literature [19,20] error rates should be greater than 50%.

Moreover, being an online experiment, it is important to control for participant’s performance during the task. To this end, reaction time measures in the recognition phase were collected as a control measure. These response times served to assess the participant’s level of engagement in the task. Participants with a mean response time faster or slower than 2.5 standard deviation of the mean group were considered outliers and removed from the analysis. Additionally, although previous studies did not measure response time, we aimed to explore whether participants were slower selecting incorrect than correct faces as well as whether response time differences were revealed for incorrect ingroup face selection (i.e., same-language errors) as compared to incorrect outgroup face selection (i.e. different-language errors).

#### Predictions

Assuming that linguistic categorization is an automatic process, we predict more same-language errors than different-language errors. That is, when participants make an error attributing a statement to a speaker, they are expected to be more likely to choose a speaker of the same language. In addition, based on a previous study [32], we predict a positive correlation between the Relative Use Index covariate and categorization.

### Results

From the 50 participants that performed the experiment, one participant with a *perceived proficiency* lower than 6 in Basque was excluded. The mean Relative Use Index was 0.21, indicating that participants used more Spanish than Basque in their daily activities and social communications. See Table 1 for participant descriptive variables. In the Research Report Protocol we declared that we would only select participants with neutral or positive *identification* towards both groups of their communities (i.e., mean score > 3). This criterion would have forced us to remove 29 out of 50 participants due to low identification with the Spanish group. Instead of removing these participants, we decided to keep them in the analysis and to perform an additional analysis to assess whether the *group identification* variable modulates the language categorization effect based on language. In order to be consistent, the same decision was taken for the analysis of data in Study 2 (see below).

**Table 1:** Mean participants descriptive variables for Study1.

Age	Group identification Spanish	Group identification Basque	Relative Use Index	Proficiency Spanish	Proficiency Basque
24.58 (8.01)	2.47 (1.24)	5.55 (1.42)	0.21 (0.50)	9.13 (0.95)	8.87 (1.05)

Standard deviations are reported in parentheses.

#### Language categorization

**Fig 2:**
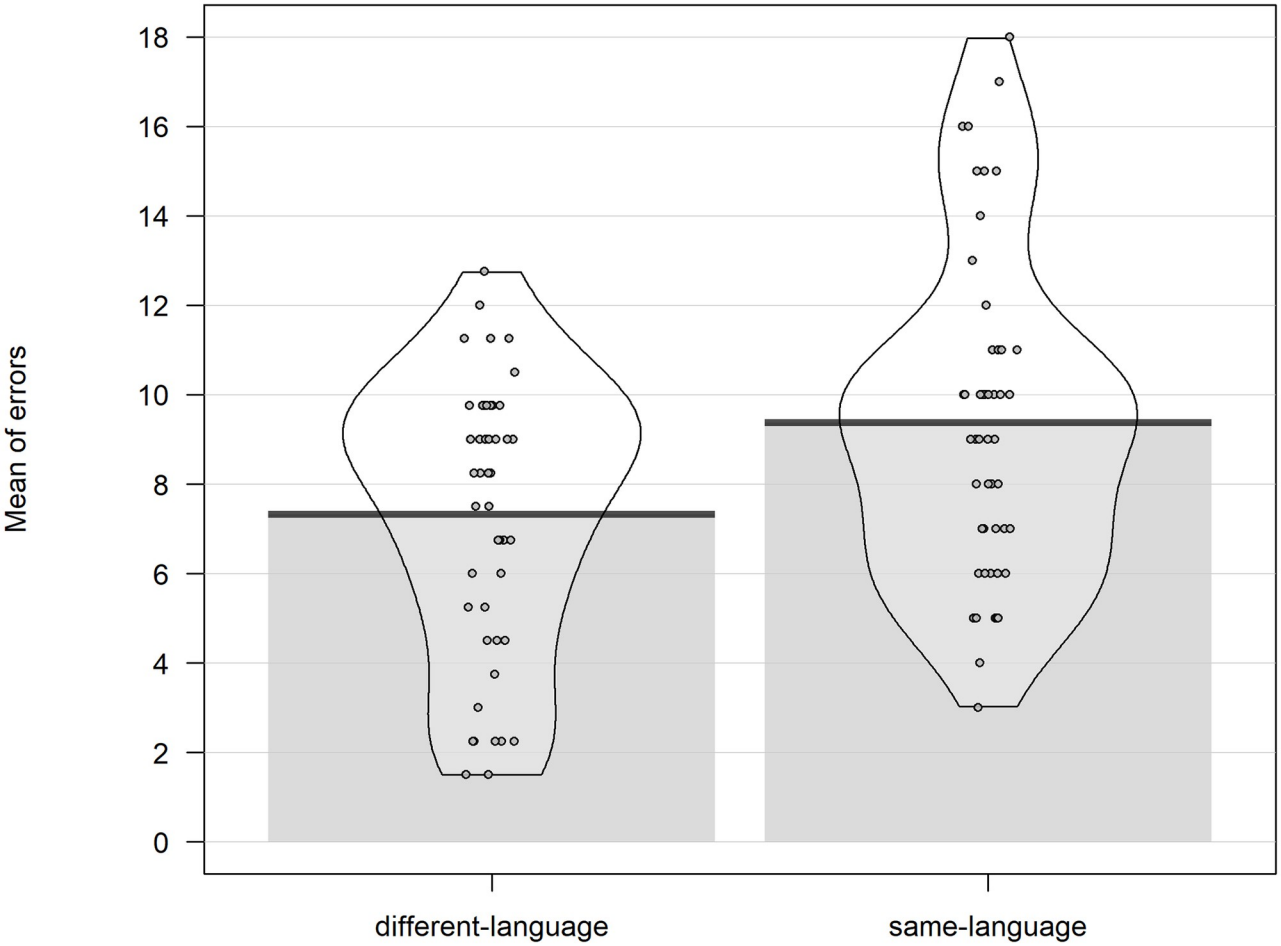
Language categorization. Mean of errors split by type of error for Study1.

Participants made an average of 19.12 total errors (SD = 3.84) out of 24 responses, making a mean error rate of 79%. The paired t-test showed that participants made significantly more same-language errors (9.36, SD = 3.62) than different-language errors (7.31, SD = 3.06; t(48) = 2.360, p = .022). See Fig 2.

The Relative Use Index was introduced as a fixed effect in a linear model with the difference between same-language and different-language errors on a participant basis as dependent variable. The effect of Relative Use Index was not significant (SE = 1.78, t = 0.06, p = .95). We note that this type of analysis differed from what we declared in the protocol, where it was proposed to add the Relative Use Index as a covariate in the paired t test.

The same type of analysis was done with the *group identification* scales. Specifically, the Spanish and the Basque group identification scales were included as fixed effects in a linear model with the difference between same-language and different-language errors on a participant basis as dependent variable. None of the scales yielded significant effects (Spanish group identification: SE = .76, t = .50, p = .61; Basque group identification: SE = .67, t = 1.11, p = .27). In a further analysis we explored the Language effect on those participants who showed a score greater or equal to 3 in both group identification scales. The paired t-test showed that participants made more same-language errors (9.45, SD = 3.06) than different-language errors (7.57, SD = 2.46). This difference was however not significant (t(19) = 1.69, p = .11), probably due to the small sample size, 21.

#### Reaction time (RT) analysis

**Fig 3:**
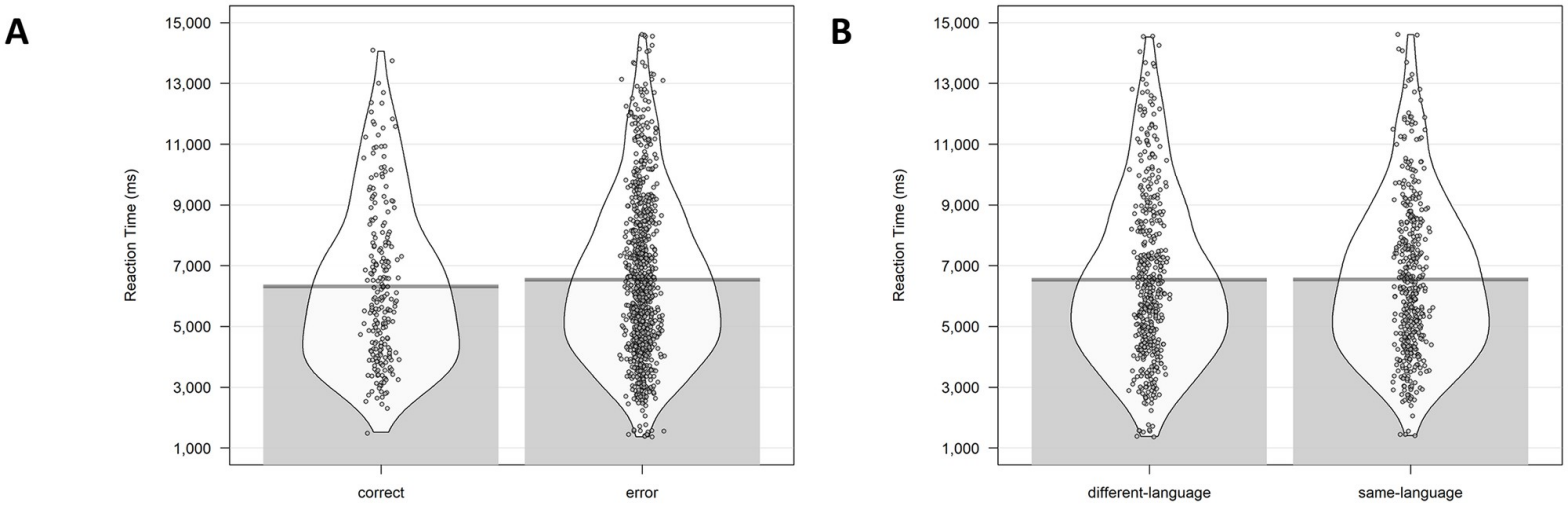
Analysis on Reaction Time for Study 1. (A) RT results between corrected answers and errors. (B) RT results between same-language and different-language errors. RTs in the figure are not transformed.

Linear-mixed effects regressions were performed on the reaction times using the lme4 package [48]. In the mixed model, the factor Response Type (correct, error) was introduced as fixed effect, and Participant and Item as random effects. As the data were not normally distributed, we used the Box-Cox test [49], using the function boxcox in the package “MASS” [50] to estimate the most appropriate transformation for the data to reduce skewedness and approximate a normal distribution. Participants were faster selecting the correct response compared to when an incorrect choice was performed (SE = .38, t = 2.88, p = .004, See Fig 3A). In a second level of analysis we tested whether there was a difference between RTs to same-language and different-language incorrect choices. No significant differences emerged (SE = .35, t = .54, p = .59, See Fig 3B).

### Study 2: Italian-Venetian bilinguals

### Materials and methods

#### Participants

68 Italian-Venetian bilingual participants took part in Study 2 (27 female). All participants were required to give written informed consent.

#### Materials

The same eight gray-scale photographs of male Caucasian faces as in Study 1 were used in Study 2. Twenty-four non-autobiographical sentences were created and then recorded in Italian and Venetian (*Il pane fresco è finito*—*El pan fresco l’è finio;* “The fresh bread is finished”, in Italian and Venetian, respectively) using the software Audacity (v 2.0.3). Sentences’ word length did not diverge between Italian [mean = 5.45 words, range = 4–8] and Venetian [mean = 5.58 words, range = 4–8] (t < 1). Four male native Italian speakers and four male native Venetian speakers recorded the sentences. Recording durations for sentences in Italian [mean = 2.01 seconds, range = 1.44–2.52] and Venetian [mean = 1.91 seconds, range = 1.35–2.79] did not differ (t(46) = 1.01, p = .31). The final design and list were identical to Study 1.

#### Procedure

Identical to Study1.

#### Methodology for data collection

Identical to Study 1. Participants were recruited through a ‘snowball’ procedure using social media.

#### Methodology for analysis

Identical to Study1.

#### Predictions

Identical to Study1.

### Results

From the 68 participants that performed the experiment, 8 participants with *perceived proficiency* lower than 6 in Venetian were excluded. The mean Relative Use Index was 0.59, indicating that participants used more Italian than Venetian in their daily activities and social communications. One participant whose mean RT was slower than 2.5 standard deviation of the group mean was excluded from the analysis, so that the final analysis included 59 participants. See Table 2 for participant descriptive variables.

**Table 2:** Participant descriptive variables for Study2.

Age	Group identification Italian	Group identification Venetian	Relative Use Index	Proficiency Italian	Proficiency Venetian
37.95 (14.29)	4.64 (1.39)	4.97 (1.59)	0.59 (0.37)	9.38 (0.86)	8.28 (1.21)

Standard deviations are reported in parentheses.

#### Language categorization

**Fig 4:**
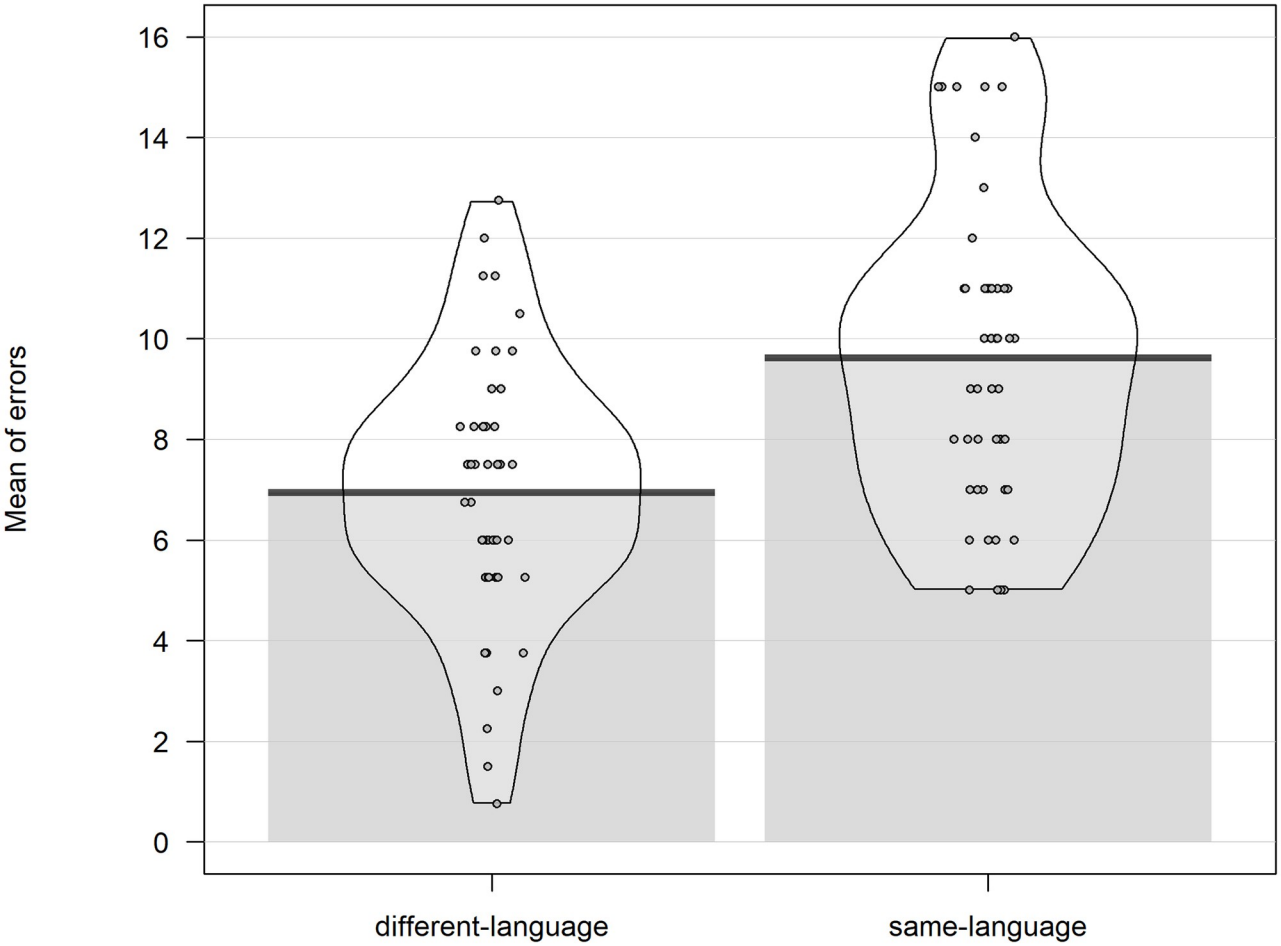
Language categorization. Mean of errors split by type of error for Study2.

Participants made an average of 19.13 total errors (SD = 3.24) out of 24 responses, making a mean error rate of 80%. The paired t-test showed that participants made significantly more same-language errors (9.54, SD = 3.11) than different-language errors (6.94, SD = 2.54, t(58) = 4.01, p < .001). See Fig 4.

The effect of the Relative Use Index and the effect of group identification scale were analysed as in Study 1. Neither the effect of Relative Use Index (SE = 1.76; t = -1.01; p = .31) nor the effects of group identification scales were significant (Italian group identification: SE = .47, t = -1.39, p = .17; Venetian group identification: SE = .41, t = -.27, p = .79).

#### Reaction time (RT) analysis

**Fig 5:**
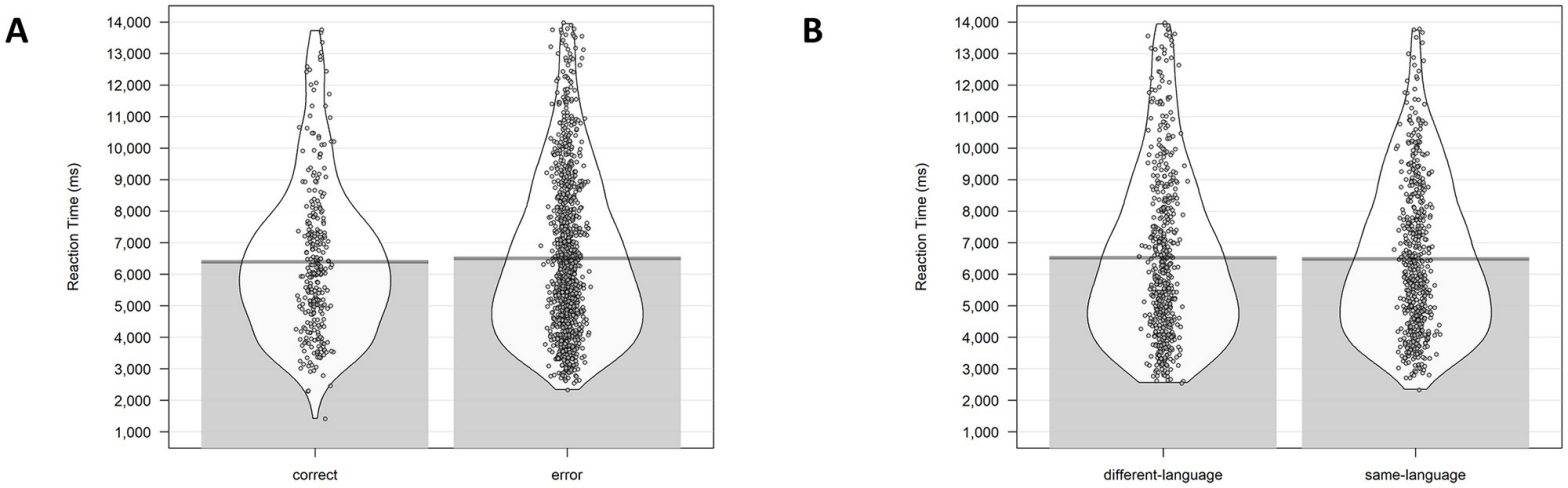
Analysis on Reaction Time for Study 2. (A) RT results between corrected answers and errors. (B) RT results between same-language and different-language errors. RTs in the figure are not transformed.

The same analysis performed in Study 1 were used in Study 2. No RT difference emerged for selecting the correct compared to the incorrect response (SE = .026, t = 1.43, p = .15, See Fig 5A). As in Study 1, no difference between RTs to the same- and different-language incorrect responses was found (SE = 0.02, t = -0.01, p = .99, See Fig 5B).

## General discussion

It has been shown that people use speakers’ language as a cue for social categorization. In two studies we examined whether bilingual participants who daily use both their languages in several contexts within their community still use language for categorizing speakers. Two groups of bilinguals were involved: Spanish/Basque speakers in Study 1 and Italian/Venetian speakers in Study 2. Using the memory confusion paradigm, we first exposed bilingual participants with eight male faces, half producing statements in one language and the other half in another language. At the test phase participants were required to identify which speaker produced each statement.

In both studies, we showed that participants were more likely to confuse faces from the same-language group than from the different-language group. These findings clearly indicate that language is used as a social cue to categorize other individuals’ faces even within bilingual communities, where language does not discriminate between social groups.

A second goal of the present research was to explore whether the categorization based on language is modulated by the degree of social interaction within each language. That is, whether the amount of time participants interact in each of the two languages is a factor modulating their categorization effect. Our results reveal that the amount of language interaction, as measured by the participants’ Relative Use Index, is not a critical factor determining the categorization effect. Furthermore, we explored whether the *group identification* towards one language identity or the other language identity modulates the categorization. Once again, our results reveal that group identity does not have an effect on the categorization based on language.

In sum, we show that the categorization effect is also present in communities in which the language is not critical for categorizing people, since we have generalized and replicated the effect with two bilingual communities where both languages are used in a daily basis. Interestingly, this happens when both languages are officially recognized, as Basque and Spanish in Study 1, and also when bilinguals use an official language and a non-official regional language, as Italian and Venetian in Study 2. Still, there are interesting questions to be addressed, such as if the same result should be obtained in those bilingual communities where individuals are classified into different groups because of the social-economic status associated to the language they used. For example, in India, English is recognized as the second official language along with Hindi. Critically, the colonial association of English with power, health and social-economic status continue to hold to date [51].

In conclusion, to the best of our knowledge, this is the first evidence showing language categorization effects in bilingual communities. Past studies investigating the role of language as a cue for social categorization have used two languages (or accents) that belonged to two different sociolinguistic contexts [19,23]. The use of bilingual communities is critical to determine whether categorization based on language is an automatic phenomenon. Our results suggest that this is the case.
